# Mild-to-moderate iodine deficiency among pregnant women in Ireland: data from a large prospective pregnancy cohort

**DOI:** 10.1007/s00394-025-03692-z

**Published:** 2025-05-09

**Authors:** Lisa Kelliher, Mairead E. Kiely, Jillian R.-M. Browne, Yvonne C. O’Callaghan, Áine Hennessy

**Affiliations:** 1 Cork Centre for Vitamin D & Nutrition Research, School of Food and Nutritional Sciences, University College Cork, Cork, Republic of Ireland; 2 INFANT Research Centre, University College Cork, Cork, Republic of Ireland; 3 School of Food and Nutritional Sciences, University College Cork, Cork, Republic of Ireland

**Keywords:** Maternal iodine status, Urinary iodine concentration (UIC), Pregnancy

## Abstract

**Purpose:**

Adequate maternal iodine status is essential for healthy foetal brain development. There are no current data on maternal iodine status in Ireland. The aim of this study was to conduct the first large-scale assessment of maternal iodine status in Ireland and identify its sociodemographic determinants using data from a large prospective pregnancy cohort.

**Methods:**

Participants were nulliparous females (*n* = 1509) recruited at Cork University Maternity Hospital, Cork, Ireland. Clinical and questionnaire-based assessments were carried out and spot urine samples were collected throughout pregnancy. Urinary iodine concentration (UIC) at 11 and 15 weeks of gestation was quantified using the Sandell–Kolthoff colorimetric method. UIC was corrected for urinary creatinine (measured via Jaffe assay), expressed as I: Cr ratio. Linear and logistic regression were performed to identify non-dietary determinants of iodine status in early pregnancy.

**Results:**

Median (IQR) UIC at 11 and 15 weeks of gestation were 128 (76, 201) and 125 (74, 208) µg/L, respectively, indicating mild-to-moderate iodine deficiency during pregnancy at both timepoints. Iodine-containing supplement use, winter season, BMI, age and education were predictors of I: Cr < 150 µg/g.

**Conclusion:**

This first large-scale investigation of maternal iodine status in Ireland highlighted sub-optimal status in pregnancy.

**Supplementary Information:**

The online version contains supplementary material available at 10.1007/s00394-025-03692-z.

## Introduction

Iodine is required for the synthesis of thyroid hormones, triiodothyronine (T3) and thyroxine (T4), which function in organ growth and development and play an important role in neuronal proliferation and migration in the developing foetal brain [1, 2]. During pregnancy, iodine requirements are increased by approximately 50% to support enhanced maternal T4 synthesis to maintain an adequate supply of thyroid hormones to the developing foetus in early gestation, to deliver an adequate supply of iodine to the foetus in later pregnancy and due to increased renal iodine clearance, placing pregnant women at a higher risk of deficiency than the general population [3]. While the impacts of moderate-to-severe deficiency on maternal and infant health outcomes are well described (increased risk of miscarriage and stillbirth [4], cretinism, neonatal hypothyroidism, impaired growth, goitre and infant mortality [5]), the adverse effects of mild-to-moderate iodine deficiency are less clear. Maternal iodine status has been associated with child neurological outcomes in some, but not all studies [6, 7].

Urinary iodine concentration (UIC) is a sensitive marker of recent dietary intake and the World Health Organization (WHO) recommends assessing population iodine status by measuring UIC from spot urine samples using adequacy thresholds of ≥ 100 µg/L and ≥ 150 µg/L for general and pregnant populations, respectively [8]. As spot UIC is subject to high day-to-day variability, it is not appropriate for classifying individual iodine status [9].

Globally, the number of iodine deficient countries, identified on the basis of UIC in school aged children, reduced significantly between 1990 and 2023 from 113 [10] to 20 [11], largely due to salt iodisation initiatives [12]. However, data in pregnant women indicate that mild-to-moderate deficiency remains prevalent [13] even in countries with established mandatory salt iodisation programmes [14, 15, 16, 17, 18].

In the United Kingdom, low iodine status in early gestation [median UIC: 91 µg/L] was reported in the Avon Longitudinal Study of Parents and Children (ALSPAC) [19]. More recently, a median UIC of 147 µg/L was observed in the UK Pregnancies Better Eating and Activity Trial (UBEAT) study of pregnant women living with obesity [20]. Previously in Norway, the prospective Norwegian Mother and Baby cohort (MoBa) study highlighted low status [median UIC: 69 µg/L] [21], and current reports from the Little in Norway (LiN) study indicate that status remains sub-optimal [median UIC: 92 µg/L] [22]. Low iodine status in early-to mid-gestation has been reported in cohorts from Sweden [median UIC: 101 µg/L] [23], and Iceland [24] [median UIC: 89 µg/L], while the Generation R Study reported iodine adequacy [median UIC: 226.9 µg/L], most likely a result of the Dutch mandatory iodised salt program [25]. Mixed findings have been reported in the United States, where median UIC of pregnant women in NHANES 2005–2010 and NHANES 2007–2014 was 129 µg/L and 144 µg/L [26, 27], respectively. However, estimates of median UIC in a large pregnancy intervention trial (Assessment of Docosahexaenoic Acid on Reducing Early Preterm Birth (ADORE), *n* = 966) [28] and in the National Children’s Study (NCS) Vanguard Study [26] indicate that iodine status is adequate (UIC: 154 and 167 µg/L, respectively).

There are few contemporary data on iodine status in pregnancy on the Island of Ireland. A longitudinal study of Northern Irish pregnant women (*n* = 241) observed low iodine status in each trimester (UIC: 73, 94 and 117 µg/L, respectively) [29]. One small-scale, cross-sectional study in early gestation (median gestation: 8 weeks) (*n* = 56) in the Republic of Ireland reported a median urinary iodine excretion (UIE) of 45 µg/L in summer and 68 µg/L in winter, corresponding to 55% and 23% of women with a UIE below a selected threshold of 50 µg/L [30].

The aim of this study was to conduct the first large scale investigation of iodine status among pregnant women in Ireland, using an extensively characterised prospective pregnancy cohort. Specifically, we aimed to analyse UIC and investigate the non-dietary determinants of UIC in Irish participants of the Improved Pregnancy Outcomes by Early Detection (IMPROvED) cohort in early pregnancy [31].

## Methods

### Participants

Participants were nulliparous women with singleton pregnancies receiving antenatal care at Cork University Maternity Hospital (CUMH), Cork, Ireland who took part in the Improved Pregnancy Outcomes by Early Detection (IMPROvED) study (*n* = 1509). Recruitment began in November 2013 and ended in June 2017. Research midwives collected information on maternal socioeconomic status, education, relationship status, lifestyle and a complete medical history, in addition to anthropometric and clinical measurements throughout pregnancy. Fasting maternal urine and sera were collected at 11- and 15-weeks’ gestation and stored at -80 °C.

### Ethical approval

The study was conducted in accordance with the Declaration of Helsinki, and ethical approval was granted by the Clinical Research Ethics Committee of the Cork Teaching Hospitals (CREC) [ECM 5 (3) 06/08/13]. Ethical approval for the secondary analysis of IMPROvED reported in this paper was granted by CREC [ECM 4 (m) 1/6/2021 and ECM 3 (cccc) 13/12/2022].

### Measurement of urinary iodine concentration (UIC) and urinary creatinine (Cr)

Urinary iodine concentration (UIC) was measured in duplicate using the Sandell-Kolthoff method according to the standard operating procedures published by the Centre for Disease Control (CDC) as part of the Ensuring the Quality of Urinary Iodine Procedures (EQUIP) program [32]. The intra- and inter-assay coefficients of variation (% CV) were 8.5% and 17.8%, respectively.

Urinary creatinine (Cr) was measured using the urinary creatinine Jaffe assay by the Randox Monaco analyser (Randox Teoranta, Meenmore, Dungloe, Co Donegal, Ireland). The intra-assay CV was < 4%.

### Statistical analysis

**Fig. 1:**
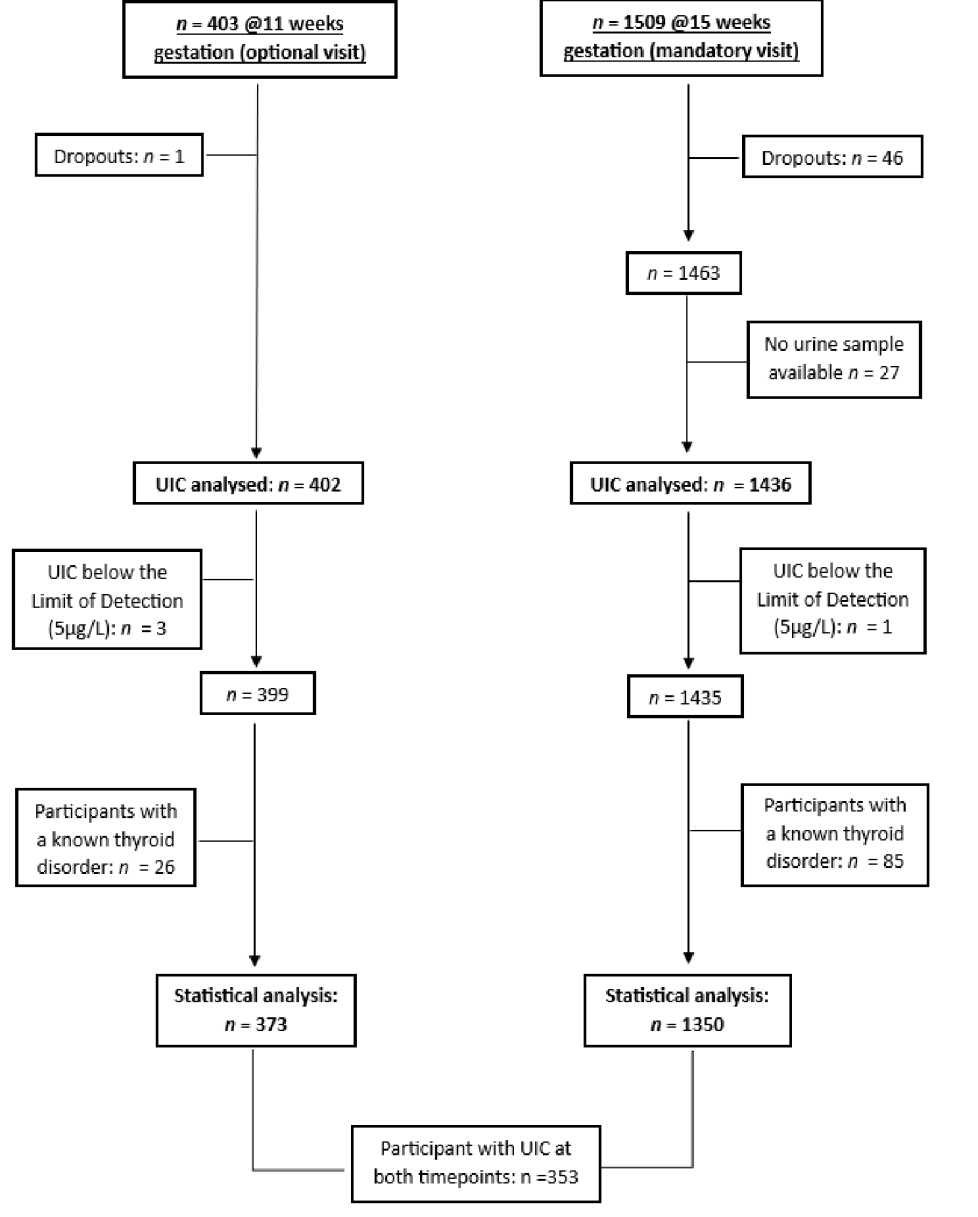
Flowchart of participants of the Improved Pregnancy Outcomes via Early Detection (IMPROvED) Study included in secondary analysis of urinary iodine concentration in early pregnancy

A total of 373 and 1350 participants were included in the analysis of UIC at 11- and 15-weeks’ gestation, respectively. Figure 1 shows a CONSORT flow diagram of participants in this secondary analysis detailing exclusion criteria (e.g. thyroid disorder, UIC below limit of detection, study drop-out). Iodine-to-creatinine ratio (I:Cr) was investigated in a sub-sample of participants (*n* = 473) whose infants entered a follow-up birth cohort, the Cork Nutrition and Development Maternal-Infant Cohort (COMBINE) [33].

Statistical analysis was conducted using IBM SPSS (Version 28, IBM Corp, Armonk NY). The distribution of data was assessed using histograms and normality tests. As distributions were right-skewed, median (IQR) UIC was reported, and differences between groups were assessed using a Mann–Whitney *U* test. An alpha < 0.05 was considered statistically significant.

Health and lifestyle determinants of iodine-to-creatinine ratio (I:Cr, µg/g) were identified in univariate linear regression. Variables with an alpha of 0.25 or predictors previously identified within the literature were entered into multivariate linear and logistic regression models to establish the determinants of I:Cr. In sensitivity analyses, interaction terms were explored between all variables within the multivariate models.

The relationship between UIC at 11- and 15-weeks’ gestation was assessed via scatterplots and Spearman’s rank correlation coefficient. A cross-classification analysis was completed to determine if the same participants had a UIC below 150 µg/L at both timepoints. UIC at 11- and 15-weeks’ gestation were split into thirds of the distribution and agreement between classifications at both timepoints was evaluated.

## Results

### Baseline characteristics of the cohort

Median (IQR) age was 31 (28, 33) years and 85% were ≤ 35 years of age. Mothers were predominantly white European (98%), employed (93%), married or in a relationship (93%) and 71% were university educated. 44% had a household annual income exceeding €64,000. Over half of the cohort had a BMI < 25 kg/m^2^ (56%). At the first study visit, 67% reported consuming an iodine-containing supplement, and 8% and 3% continued to smoke cigarettes and consume alcohol during their pregnancy, respectively (Table 1).

### Urinary iodine status in early gestation

**Fig. 2:**
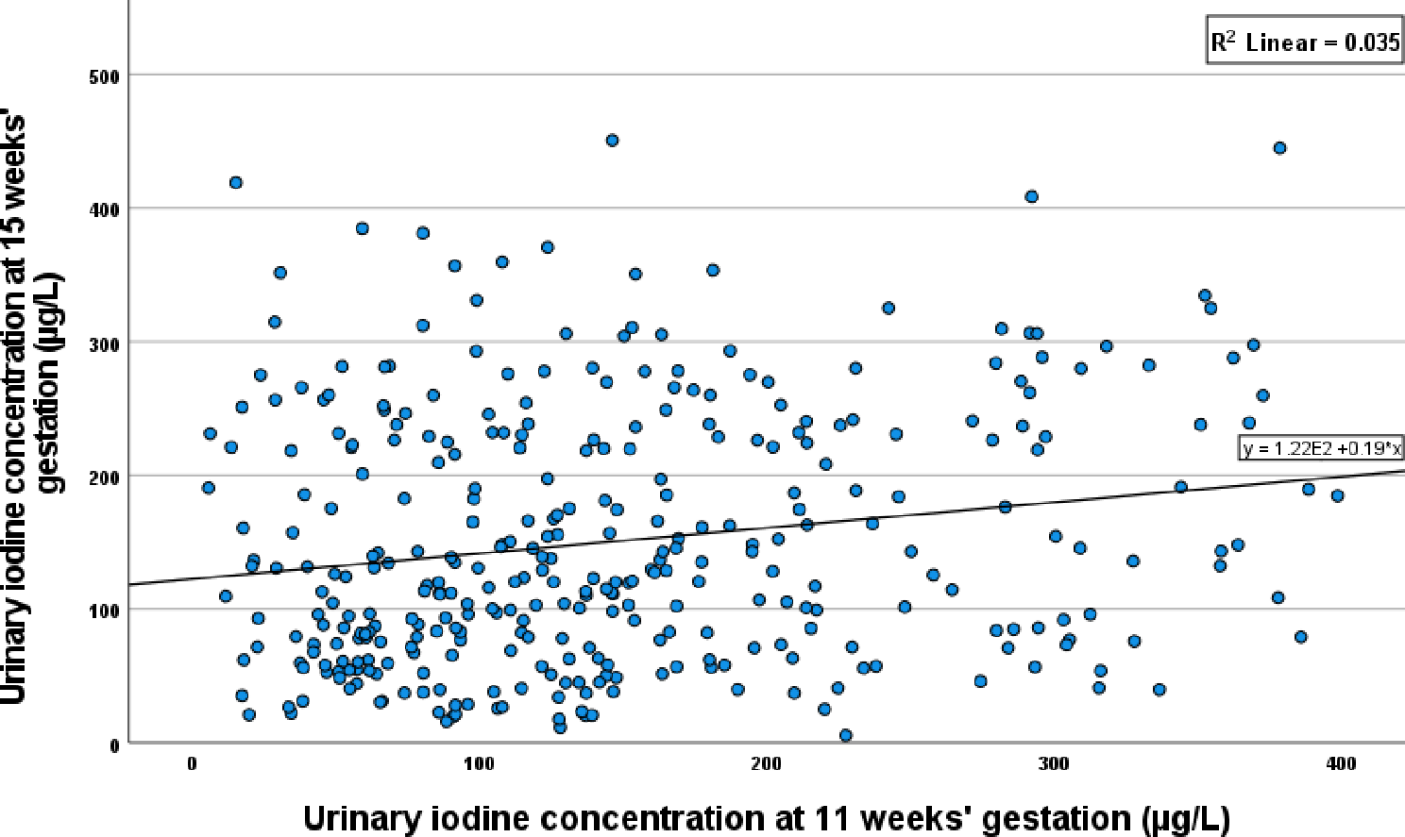
Scatterplot of urinary iodine concentration at 11- and 15-weeks’ gestation (µg/L)

Median (IQR) UIC at 15 weeks’ gestation was 125 (74, 208) µg/L, with significantly higher UIC observed in iodine-containing supplement users compared with non-users, women sampled in winter compared to those sampled in summer, and in women who were not in employment compared to women who were employed (Table 1). In the sub-sample of participants who completed the 11-week visit, median (IQR) UIC was 128 (76, 201) µg/L (Supplementary Table 1). UIC at 11- and 15-weeks’ gestation were poorly correlated (*r* = 0.181, *P* < 0.001) (Fig. 2) while 44 and 38% of participants were classified in same or adjacent category of UIC, respectively.

### Iodine creatinine ratio (I: Cr) and sociodemographic and lifestyle determinants at 15 weeks’ gestation

Median urinary I:Cr in the subsample of 443 women whose infants enrolled in the COMBINE cohort was 212 (123, 333) µg/g. Median (IQR) UIC was not significantly different in these women compared to those without an I:Cr measurement [121 (136) versus 129 (135) µg/L, respectively (*P* = 0.187)]. Within fully adjusted multivariable linear regression models, university education [β (95% CI): 64 (7, 120) µg/g, *P* = 0.027], BMI [β (95% CI): -7 (-12, -2) µg/g, *P* = 0.0008], iodine-containing supplement use [β (95% CI): 74 (26, 122) µg/g, *P* = 0.002] and winter UIC sampling [β (95% CI): 59 (15, 102) µg/g, *P* = 0.008] were significant predictors of I:Cr. Iodine-containing supplement users, participants sampled in winter and university educated participants had 71%, 57% and 62% lower odds of I: Cr < 150 µg/g, respectively. Every year increase in age was associated with 7% lower odds of I:Cr < 150 µg/g; while every kg/m^2^ increase in BMI resulted in 6% higher odds of I:Cr < 150 µg/g. (Table 2.) No significant effect modification was observed in sensitivity analysis using interaction terms between each sociodemographic factor.

## Discussion

In this first large-scale investigation of the iodine status of Irish pregnant women in early gestation, median (IQR) UIC [125 (74, 208) µg/L] indicated mild-moderate iodine deficiency. Median (IQR) UIC in the current study was similar to other large pregnancy cohorts from the UK [91 (54, 143) µg/L], Norway [92 (59,140) 92 µg/L], Turkey [94 (52, 153) µg/L], Spain [123 (73, 208) µg/L], Sweden [101 (95, 108) µg/L] and Iceland [89 (42, 141) µg/L)] [22, 23, 24, 34, 35]. By contrast, results from large nationally representative cohorts indicated widespread iodine sufficiency in the Netherlands [226.9 µg/L (90% range: 55.2- 732.6)] and India [178 µg/L], where mandatory salt iodisation is implemented and coverage is adequate (60% and 78%, respectively) [25, 36].

Diet is the main predictor of iodine status, with cows’ milk and dairy products making a significant contribution in both pregnant [24, 37, 38] and non-pregnant populations [39, 40]. As dietary data were not available for participants of the IMPROVED study, this study therefore focused on the sociodemographic determinants of I:Cr. In fully adjusted models, iodine-containing nutritional supplement use, education, BMI, winter UIC sampling and age were significant predictors of I:Cr < 150 µg/g.

The prevalence of iodine-containing supplement use in the current study was high (67%, median dose: 150 µg). We found that supplement users had a 26 µg/L higher UIC than non-users, even though the median UIC of supplement users was only 135 µg/L. This is similar to observations in Norwegian and Swedish pregnancy cohorts where supplement users (33% and 35% of participants, respectively) had higher UIC than non-users [120 vs. 83 µg/L and 149 vs. 85 µg/L, respectively], but both groups demonstrating median UIC below the threshold for pregnancy [22, 23]. Compared to the Spanish INMA cohort [34, 41] (which assessed UIC across three regions: Sabadell, Valencia, Gipuzkoa), supplement use in our study was higher than Sabadell (11%) and Valencia (49%), but lower than the Gipuzkoa region (93%) where there is a policy for iodine supplementation during pregnancy. Gipuzkoa, which also had 47% iodized salt consumption was the only region with adequate status [median UIC (IQR): 168 (108, 272) µg/L] within the INMA cohort. The Sabadell region which had the lowest UIC [94 (57, 151) µg/L], also had the lowest supplement use (11%) and iodized salt consumption (26%) of the three regions. Valencia, despite 64% iodized salt consumption and supplement use in one-in two women, had inadequate UIC [134 (80–218) µg/L]. This indicates the potential for a supplementation policy to improve iodine status in women and yet highlights that supplements alone may not achieve enough impact to ensure iodine adequacy in this vulnerable group. Australia has adopted a dual approach with mandatory fortification of bread with iodized salt and a supplementation recommendation (150 µg/d) from the point of planning and for the duration of pregnancy and breastfeeding [42]. A study of South Australian pregnant women 2 years post-fortification [43] reported adequate median UIC (159 µg/L); and found the UIC of those consuming an iodine containing supplement ≥ 150 µg/d was higher than non-supplement users (221 µg/L vs. 159 µg/L) and (187 µg/L vs. 141 µg/L) at 20- and 28-weeks’ gestation, respectively. The authors concluded maintaining adequate iodine status in pregnancy proves difficult without supplementation even after fortification of a staple food source. A 2019 study in pregnant Tasmanian women demonstrated the protective effect of iodine supplement use, where median UIC was higher in supplement users (67%) than in non-users (155 µg/L versus 113 µg/L) [44]. The majority of supplement users only commenced use in pregnancy (72%) and this sub-group had lower status (137.5 (82.5–233.5) µg/L) than the group who adhered to the recommendation to begin use prior to pregnancy (28%) (196 (98–315) µg/L).

With regards to the supplementation practices of the cohort, our previous analysis showed 30% used multivitamins (of which 42% were prenatal supplements) prior to pregnancy [45]. Of the prenatal supplements recorded, 80% contained iodine (median dose: 150 µg/d). Women who were older, non-smokers and university educated were significantly more likely to use multivitamins prior to pregnancy, likely signalling an intention to conceive. In Ireland and the UK, low iodine status has been linked to poor knowledge on iodine as a nutrient and on its importance in pregnancy [46]. Strategies to improve public awareness of iodine should be implemented by public health agencies and clinicians.

Notably, we observed winter sampling was associated with higher iodine status at 15-weeks’ gestation, independent of nutritional supplement use. In sensitivity analysis, no significant effect modification was observed on examining interaction terms between season of sampling and supplement use or with any other variables. Nationally representative data in Irish women of childbearing age show sub-optimal dietary iodine intakes (median: 104 µg/d) [39]. As the dietary sources of iodine change little in pregnancy [37], it is likely that dietary intakes are insufficient in our cohort. Though no dietary data were collected for the IMPROvED study, this finding likely points towards cow’s milk consumption as an important predictor of status in the cohort. In Ireland, cow’s milk contributes 28–37% to iodine intake of women of childbearing age [39, 47] and Irish pregnant women (30%) [37]. The iodine content of milk varies considerably based on season of production [48, 49, 50], with higher levels observed in winter when cows are fed iodine fortified feed compared to pasture fed in the summer (16 vs. 62 µg/100 g) [51]. These fluctuations have been shown to impact the iodine intake and status of Irish adults [39] and toddlers [52].

Interestingly, we identified sociodemographic predictors of I:Cr in this high-resource cohort. Women who attained university education had a 66 µg/g higher I:Cr and 3-fold lower odds of I: Cr < 150 µg/g. This is a relatively novel finding, as previous studies have not reported any meaningful association between education level and iodine status [28, 53, 54]. In our study, age was associated with I:Cr; every year increase resulted in 7% lower odds of I:Cr below 150 µg/g. Similarly, an analysis by Dineva and colleagues on determinants of iodine status in the Spanish *INMA*, Dutch *Generation R* and British *ALSPAC* studies highlighted maternal age as a determinant of iodine status in all three cohorts [54].

BMI was also a significant predictor of iodine status, with a 6 µg/g lower I:Cr for every kg/m^2^ increase in BMI. This is in line with findings of Dineva et al. [54] where a negative association between BMI and I:Cr was observed in the INMA, Generation R and ALSPAC cohorts. Others reported no association of body weight with UIC [20, 55, 56]. Obesity can have implications for thyroid function thus has been linked to higher TSH levels and lower UIC status in school aged children [57, 58]. However, indices of body composition were not available to explore if those with an overweight or obese BMI also had excess adiposity. Taken together, the association between BMI and iodine status is unclear and warrants further research; collection of other body composition data could facilitate the distinction between those who are classed as overweight by BMI but do not have excess adiposity.

Interestingly, at a population level the median UIC is very similar between both timepoints and 81% were classified in the same or adjacent third of UIC.

### Strengths and limitations

A strength of this study is the large sample size (*n* = 1350). It has been proposed that a sample of 500 is needed to assess the iodine status (UIC) of a population, with 5% precision [59] and intra-individual variation in UIC levels out in large populations [60]. UIC is reflective of recent intake and is useful in the assessment of population iodine intake. Spot urine collections are efficient and effective at a population level and with sufficient samples sizes tend to correlate well with 24-hour collections [60, 61]. However, UIC is not suitable as a marker of individual iodine status. Assessing I:Cr is useful as pregnancy cohorts are more susceptible to variation in urine dilution due to increased glomerular filtration rate. Relying on UIC alone could lead to overestimation of deficiency and it has been suggested that reporting I:Cr is suitable for homogeneous population groups [62]. The current study is limited by the absence of dietary intake data as these were not collected. Pregnancy cohorts should collect dietary intake data where possible, as many risk factors for perinatal health are underpinned by nutritional factors.

The effects of mild-to-moderate deficiency are unclear; however, recent studies indicate an association between maternal iodine deficiency and adverse cognitive outcomes in infants, such as lower working memory [63], reading accuracy and comprehension [19, 64], cognitive scores [34], impaired receptive and expressive language skills in toddlers [65].

The iodine status of Irish pregnant women is of public health concern, considering the low status we observed in the current study, the absence of published recommendations for iodine supplementation [66], no mandatory fortification programme, limited availability of iodised salt [67], and low public awareness of the importance of iodine in pregnancy [46]. While the WHO recommends a supplement of 250 µg/d for pregnant women in areas where access to iodised salt is low (< 20%) [68], the American Thyroid Association recommends all pregnant women, regardless of access to iodised salt, take an iodine containing multivitamin supplement (150 µg/L) [54]. However, the data on the effect of iodine supplement use on neurodevelopmental outcomes is mixed. Reports from the Little in Norway cohort (LiN), the Norwegian Mother and Child Cohort Study (MoBa) and the Spanish Environmental Childhood cohort (INMA) found no protective effect of supplements on infant neurodevelopmental outcomes at 18 months, 3, and 4–5 years, respectively [18, 48, 55]. These studies highlight how supplementation during pregnancy may not correct for insufficient peri-conceptional status within the critical period of neurodevelopment. A recent systematic review and meta-analysis identified the absence of robust evidence to support an iodine supplementation recommendation, particularly pertaining to timing of intake, dosage, and supplement type, in regions of mild-to-moderate deficiency [69].

In conclusion, our findings from this large cohort indicate that iodine intake in pregnant women in Ireland during early- to mid-gestation is of public health concern. Further research is required to determine the specific consequences of mild-to-moderate iodine deficiency on infant neurodevelopment and cognition, and there is a need for randomized control trials examining the effects of supplementation during pregnancy.

**Table 1:** Urinary iodine concentration (UIC) (µg/L) at 15 weeks of gestation across demographic and lifestyle characteristics in participants of the improved pregnancy outcomes by early detection (IMPROvED) study (*n* 1350)

Variable	*n* (%)	UIC (µg/L)		*P*
		Median	(IQR)	
**UIC (µg/L)**	1350	125	(74, 208)	
**Age (years)**				
< 35	1147 (85.0)	126	(75, 205)	0.583
≥ 35	203 (15.0)	123	(69, 231)	
**Ethnicity**				
White European	1319 (97.7)	126	(74, 208)	0.765
South Asian or East Asian	26 (2.3)	125	(73, 186)	
**Level of education**				
University	957 (70.9)	129	(73, 210)	0.265
Secondary	393 (29.1)	118	(75, 203)	
**Relationship status**				
Married/in relationship	1260 (93.3)	126	(73, 208)	0.754
Single	90 (6.7)	124	(77, 230)	
**Employment status**				
Employed	1251 (92.7)	124	(73, 206)	0.046
Unemployed	99 (7.3)	143	(86, 246)	
**Annual household income**				
< € 21,000	121 (9.4)	123	(82, 217)	0.865
≥ € 21,000	1169 (90.6)	127	(74, 208)	
< € 64,000	702 (54.4)	122	(74, 194)	0.068
≥ € 64,000	588 (45.6)	132	(75, 224)	
**Type of maternity health care**				
Public	1307 (96.8)	125	(73, 208)	0.179
Private	43 (3.2)	138	(91, 233)	
**BMI (kg/m** ^**2**^ **) at 15 weeks’ gestation**				
< 25.0	759 (56.3)	129	(77, 217)	0.066
≥ 25.0	589 (43.7)	121	(70, 196)	
**Iodine-containing nutritional supplement use in the first trimester**				
Supplement user	908 (67.3)	135	(79, 220)	< 0.001
Non-supplement user	442 (32.7)	109	(65, 175)	
**Cigarette smoking prior to pregnancy**				
Smoker	374 (27.7)	122	(72, 199)	0.585
Non-smoker	976 (72.3)	127	(74, 211)	
**Cigarette smoking during pregnancy**				
Smoker	103 (7.6)	115	(68, 197)	0.396
Non-smoker	1247 (92.4)	127	(74, 209)	
**Alcohol consumption prior to pregnancy**				
Consumer	1210 (89.6)	126	(74, 208)	0.884
Non-consumer	140 (10.4)	123	(67, 215)	
**Alcohol consumption during pregnancy**				
Consumer	34 (2.5)	135	(60, 238)	0.634
Non-consumer	1316 (97.5)	125	(74, 208)	
**Season of sampling**				
Winter	735 (54.4)	136	(79, 221)	< 0.001
Summer	615 (45.6)	113	(66, 197)	

**Table 2:** Sociodemographic and lifestyle determinants of iodine-to-creatinine ratio (I: Cr) at 15 weeks’ gestation below 150 µg/g (*n* 428)

Variable	Category	*n*	OR (95% CI)	*P*
**Nutritional supplement use**	Non-supplement user	126	Reference	< 0.001
	Iodine-containing supplement user	302	0.29 (0.18, 0.46)	
**Season of sampling**	Summer	200	Reference	< 0.001
	Winter	228	0.43 (0.28, 0.68)	
**University education**	No	106	Reference	< 0.001
	Yes	322	0.38 (0.22, 0.68)	
**Age (years)**	-	-	0.93 (0.88, 0.99)	0.014
**BMI (kg/m** ^**2**^ **) at 15 weeks’ gestation**	-	-	1.06 (1.01, 1.11)	0.027
**Cigarette smoking during pregnancy**	Non-smoker	392	Reference	0.214
	Smoker	36	1.70 (0.74, 3.95)	
**Household income (annual)**	≥ € 64,000	208	Reference	0.368
	< € 64,000	220	1.25 (0.77, 2.02)	
**Relationship status**	Married or in relationship	404	Reference	0.727
	Single	24	1.19 (0.45, 3.11)	
**OR**, odds ratio; **95% CI**, 95% confidence intervals				

## Electronic supplementary material

Below is the link to the electronic supplementary material.

Supplementary Material 1
